# Chronic Lung Allograft Dysfunction Is Associated with Significant Disability after Lung Transplantation—A Burden of Disease Analysis in 1025 Cases

**DOI:** 10.3390/arm91050033

**Published:** 2023-10-12

**Authors:** Roland Diel, Susanne Simon, Jens Gottlieb

**Affiliations:** 1 Institute for Epidemiology, University Medical Hospital Schleswig Holstein, Campus Kiel, Niemannsweg 11, 24105 Kiel, Germany; 2 Lung Clinic Grosshansdorf, Airway Disease Center North (ARCN), German Center for Lung Research (DZL), 22949 Großhansdorf, Germany; 3 Department of Respiratory Medicine, Hannover Medical School, 30625 Hannover, Germany; simon.susanne@mh-hannover.de (S.S.); gottlieb.jens@mh-hannover.de (J.G.); 4 Biomedical Research in Endstage and Obstructive Lung Disease Hannover (BREATH), German Center for Lung Research (DZL), 30625 Hannover, Germany

**Keywords:** lung transplantation, chronic lung allograft dysfunction, disability, bronchiolitis obliterans syndrome, disability-adjusted life years, patient outcome assessment

## Abstract

**Highlights:**

**What are the main findings?**

- One out of four lung transplant recipients in the largest German center develop chronic lung allograft dysfunction (CLAD), which corresponds to chronic lung transplant rejection.
- Transplant patients who develop severe CLAD subsequently lose more than two disability-adjusted life years (DALYs).

**What are the implications of the main findings?**

- As CLAD disability slowly develops within a median of two years after lung transplantation, there is room for better management of CLAD.
- Beyond already existing options (azithromycin, tacrolimus), new options have to be found for the prevention of CLAD.

**Abstract:**

Background: Chronic lung allograft dysfunction (CLAD) is the leading cause of death after the first postoperative years of lung transplantation (LTx). Objective: To assess the number of disability-adjusted life years (DALYs) per patient with severe CLAD. Methods: The clinical and demographic data of patients who received their lung transplantation between 2010 and 2020 in the Hanover Medical School (Germany) were evaluated. Results: A total of 1025 lung transplant patients were followed for a median of 51 months (4.25 years); the median age at transplantation was 52.8 (interquartile range (IQR) 19) years. More than a quarter of transplant patients (271/1025 or 26.4%) developed CLAD, mostly (60%) of the bronchiolitis obliterans syndrome (BOS) phenotype. Of the CLAD patients, 99, or 36.5%, suffered from significant disability, which on average occurred after 2 years (IQR 2.55). The survival of CLAD patients with disability after transplantation was significantly lower compared to that of patients without CLAD (median 4.04 versus 5.41 years). Adjusted to the DALY estimation approach, CLAD patients lost 1.29 life years (YLL) and lived for 0.8 years with their disability (YLD), adding up to 2.09 DALYs (range 1.99–2.72) per patient. Conclusions: CLAD after lung transplantation is a major public health problem and is associated with substantial disability and costs. Further work is needed to develop therapeutic interventions that reduce its development.

## 1. Introduction

Lung transplantation (LTx) is an established treatment modality in a number of lung diseases, where it improves survival and quality of life. As activity has been increasing over the last decade, in 2012, a total of 6470 lung transplantations were estimated to have been performed worldwide: of those, 1964 were in Europe [1].

Post-transplant survival percentage reached in experienced centers is more than 90% at one year, 60–70% at five years and 40–50% at ten years [2]. Chronic lung allograft dysfunction (CLAD) is the major obstacle to the long-term success of LTx. According to registries, this disease affects up to 50% of lung transplant recipients within 5 years after transplant [3].

CLAD is not only the leading cause of death after the first postoperative year but also causes significant morbidity and has limited treatment options. Most affected patients will die from progressive respiratory failure, with a median survival of 2.5 years [4]. Chronic lung allograft dysfunction (CLAD) has two main clinical presentations with potential overlap (mixed phenotype): bronchiolitis obliterans syndrome (BOS) and restrictive allograft syndrome (RAS) [5]. RAS is characterized by restrictive lung function decline and interstitial fibrotic process. The RAS and mixed phenotype occur in approximately 30% of CLAD patients and have a worse prognosis than the BOS phenotype [6].

To assess the burden of CLAD in lung transplant patients, the determination of disability-adjusted life years (DALYs) is the most suitable instrument. A DALY consists of two additive components: years lost due to disability (YLD) and years of life lost due to premature death (YLL); thus, DALY = YLL + YLD [7]. In contrast to quality-adjusted life years (QALYs) which are simply the product of one life year gained and a constant value representing the utility of suffering from a given disease, DALYs include a discounted age-weighting function to take into account that value of life may vary with age and that future events have a lower value when aggregated to a value in the present [7]. The goal of this study was to evaluate the burden of significant disability in CLAD patients within a large cohort of lung transplant recipients, which, to our knowledge, has not yet been done before.

## 2. Methods

### 2.1. Clinical and Demographic Data Assessment

A prospective single-center cohort study was performed at the Hanover Medical School (MHH), Germany. Patients after LTx were consecutively included between 1 January 2010 and 30 June 2020. They were transplanted between 1 January 2010 and 31 March 2020 and followed for at least 3 months of follow-up to allow for enough pulmonary function tests to diagnose and classify CLAD and further investigated in the MHH outpatient clinic. As CLAD is defined by a reduction in spirometry in relation to the baseline measurement after transplantation (mean of the best two values), baseline forced expiratory volume in one second (FEV1), forced vital capacity and total lung capacity had to be measured. Patients were excluded from our study when computed tomography imaging or body plethysmography were not available or when they were not able to fill out questionnaires due to illiteracy and limited language skills. Informed consent to use patient data was obtained according to the ethics vote of Hannover Medical School (2923-2015).

Spirometry and body plethysmography were performed according to ATS/ERS standards [8,9]. CLAD was diagnosed by a persistent (at least 3-month) decline of FEV1 to 80% of baseline or below after exclusion and adequate treatment of secondary causes such as infection, acute cellular/antibody-mediated rejection, or airway stenosis according to current definitions [6]. The presence of persistent opacities on chest imaging with or without pleural changes and a loss of lung volume (total lung capacity ≤ 90% baseline) were used for case definition. The mixed phenotype was defined by persistent opacities plus FEV1/FVC < 0.7, with accompanying total lung capacity (TLC) decline, while restrictive allograft syndrome (RAS) was defined as persistent opacities and pure restriction on spirometry. Bronchiolitis obliterans syndrome was defined as obstructive physiology without opacities, with normal lung values. All other combinations of FEV1/FVC, TLC and opacities were defined as the “other” phenotype.

CLAD severity was staged according to the International Society of Heart and Lung Transplantation (ISHLT): CLAD 0: FEV1 > 80% of baseline, CLAD 1: FEV1 66–80% of baseline, CLAD 2: FEV1 51–65% of baseline, CLAD 3: FEV1 35–50% of baseline and CLAD 4: FEV1 < 35% of baseline [5]. All CLAD patients received azithromycin (250 mg 3 times per week) as standard treatment. Slowly progressive patients received add-on therapy with montelukast (10 mg once daily); rapidly progressive patients were offered additional extracorporeal photopheresis (ECP).

### 2.2. Calculation of DALYs

To assess the impact of CLAD on lung transplant patients we had to adjust the calculation of disability-adjusted life years (DALYs), measured in units of one year, to our cohort. Usually, to obtain the number of life years lost (*YLL*) due to a specific disease, the number of premature deaths is multiplied by the standard life expectancy at the age at which death occurs according to Equation (1):

(1) $$YLL=\frac{K\cdot C\cdot e^{r\cdot a}}{{\left(r+\beta \right)}^{2}}\cdot \left[\left[1+\left(r+\beta \right)\cdot a\right]\cdot e^{-\left(r+\beta \right)\cdot a}-\left[1+\left(r+\beta \right)\left(L+a\right)\right]\cdot e^{-\left(r+\beta \right)\left(L+a\right)}\right]+\frac{1-K}{r}\left(1-e^{-r\cdot L}\right)$$

with · denoting the mathematical multiplication sign, and *a* = age of death (years), *r* = discount rate (*r* = 0.03 in this study), *β* = age-weighting constant (*β* = 0.04), *K* = age-weighting modulation constant (*K* = 1.00), *C* = age-weighting scaling constant (*C* = 0.1658) and *L* = country-specific standard life expectancy at age of death (years).

In our cohort, in which standard life expectancy cannot be applied, the loss of life expectancy of patients with severe CLAD has to be determined by subtracting their remaining life expectancy from that of transplant patients without such a course. For this purpose, age immediately after transplantation and age at death were determined in each subgroup.

To determine the years lived with the disability (*YLD*), a so-called “disability weight” (*DW*) of a specific disease has to be incorporated. Currently, there is a fixed, standardized set of so-called “disability weights” (*DW*s) for a limited range of medical conditions, published at irregular intervals as the results of Global Burden of Disease (GBD) projects since 1996 [10]. They are estimated by pairwise comparisons addressing which of two individuals in different health states is healthier than the other. As a *DW* for CLAD patients has not yet been validated, we used the clinical definitions of the most recent 2019 GBD study [11] for severe COPD without heart failure as a substitute for “significant disability”. In our view, the restrictions on mobility and independence of CLAD patients in everyday life can best be described by the physical limitations listed for that *DW*: great difficulty in walking even short distances or climbing any stairs, feels tired at rest. The corresponding value for those patients is 0.408 with a uniform distribution between 0.273 and 0.556 [12].

Equation (2) can be used to calculate the *YLD*:

(2) $$YLD=DW\cdot \left[\frac{K\cdot C\cdot e^{r\cdot \alpha }}{{\left(r+\beta \right)}^{2}}\cdot \left[\left[1+\left(r+\beta \right)\cdot \alpha \right]\cdot e^{-\left(r+\beta \right)\cdot \alpha }-\left[1+\left(r+\beta \right)\left(T+\alpha \right)\right]\cdot e^{-\left(r+\beta \right)\left(T+\alpha \right)}\right]+\frac{1-K}{r}\left(1-e^{-r\cdot T}\right)\right]$$

Besides the *DW*, it also utilizes the duration of living with the impairment (*T*) and the age of onset of disability in years (α). As significant disability in CLAD patients does not occur immediately, but develops after a latency period, the median value of that gap before death had to be assessed.

To estimate the number of patients who fulfilled the definition of “severe disability”, an expert panel decided to use a standardized questionnaire which included three options for describing and valuing health on every visit. The first one was the EQ-5D, which defines health in terms of 5 dimensions: mobility, self-care, usual activities, pain/discomfort and anxiety/depression [13]. The EQ-5D is a generic, standardized instrument for measuring health-related quality of life. Each dimension has 5 possible responses, from no problems to extreme problems. In order to be able to show the differences between these five responses as much as possible, each dimension was scored as 0 to 20 points in 5-point increments, leading to a total score between 0 (lowest health status) and 100 (perfect health). The EQ-5D-5L score was calculated as follows: subscores were equally summed up from 0 (lowest) to 20 (highest) in 5-point increments to form a total score between 0 (low) and 100 (highest) points. Based on pre-validations on a representative subsample of lung transplant patients in 2009 (unpublished data), significant disability was defined as patients reporting a persistent total EQ5D score of less than 60 points. The score also considers that patients who rapidly progress to CLAD, i.e., have an FEV loss of more than 100 mL per month, may experience more dyspnea, which might affect the individual perception of disability. To more concretely describe the limitation of physical resilience that takes on a special role in lung transplantation [14] beyond the generic category of “mobility”, we also asked for about the number of stairs that could be climbed by the patient and whether a rollator or wheelchair was continuously necessary. If there was need for a wheelchair or a rollator or inability to climb one flight of stairs, the patient was considered significantly diseased, even when the number of points in the EQ5D score was equal to or more than 60 points.

### 2.3. Statistics

Statistical analysis was performed with metric variables expressed as medians, the interquartile range (IQR), i.e., the region between the 75th and 25th percentile, and categorical variables as absolute numbers and percentage of data entries. Univariate analyses were performed using the Mann–Whitney test for continuous variables and chi-square test for categorical variables. Survival analysis was performed using the Kaplan–Meier method, and the differences in survival outcomes between groups were compared using the log-rank test. Binary logistic regression analyses were conducted with significant disability as the outcome variable. The level of significance was set at ≤0.10 to include variables identified by univariate analysis between groups. Data were analyzed as observed, without the imputation of missing values.

## 3. Results

**Figure 1:**
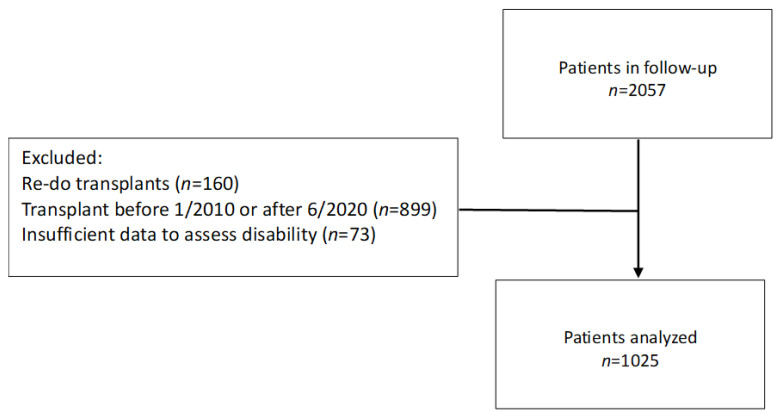
Flowchart of patient identification.

A total of 1025 patients with complete data were identified (Figure 1), 489 females (47.7%) and 536 males (52.3%). Patient demographics are displayed in Table 1. The majority of patients received a bilateral transplantation, and the median follow-up was 51 months or 4.25 years. The median age at transplantation was 52.8 (25th percentile 40.0; 75th percentile 58.0; IQR 18) years. The most frequent comorbidities recorded during follow-up were insulin-dependent diabetes (23%), coronary artery disease (5%) and significant kidney dysfunction (4%).

**Figure 2:**
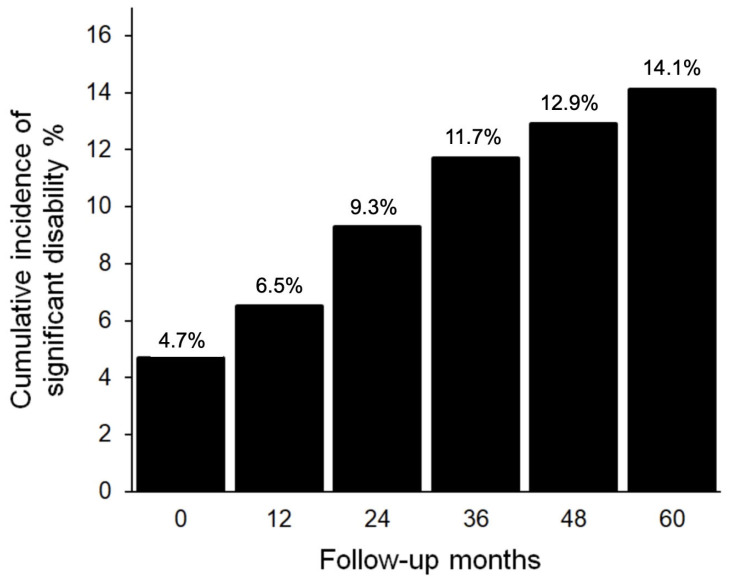
Incidence of significant disability during follow-up (patients alive).

Two hundred and twelve patients (20.7%) died during follow-up on average 2.9 years (median) after lung transplantation (25th percentile 1.7; 75th percentile 4.3; IQR 2.6). The most frequent causes of death were CLAD (41%), infections (14%) and malignancy (15%). The median age at death of all included patients was 56.6 years (25th percentile 49.8; 75th percentile 62.7; IQR 13). One hundred and seventy-one patients reported persistent significant disability during follow-up, which increased during follow-up (Figure 2). The median onset of disability was two years after LTx (25th percentile 1.1; 75th percentile 3.65; IQR 2.55). Seventy-seven deaths (31%) were preceded by significant disability.

Out of the 1025 patients, 274 (26.7%) developed CLAD during follow-up. The CLAD phenotypes were BOS, RAS or mixed and other in 157 (65%), 70 (26%) and 14 (7%) patients, respectively. Thirty-four patients developed pure RAS. In total, 171 patients reported persistent significant disability during follow-up (84 patients by EQ5D < 60 points; in addition, 41 patients by wheelchair/rollator and 46 patients by immobility criterion (inability to climb a flight of stairs)). The distribution of patients (in percent) on the individual levels of the dimensions is shown in Supplementary Materials Table S1. Four hundred and eighty-nine patients had their last visit during the pandemic (since 1 January 2020). One COVID-19 patient, who was recorded on 19 March 2020 and was included in our cohort, remained stable and did not develop CLAD during the study period.

**Figure 3:**
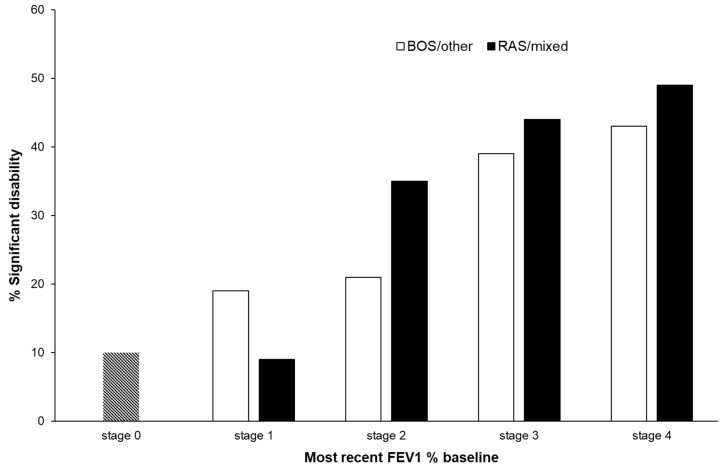
CLAD stages and significant disability.

Univariate analysis identified female gender, age, an underlying disease of COPD, pulmonal vascular disease, psychiatric illness and CLAD as risk factors for developing disability (Table 2). In multivariate analysis, just the diagnosis of CLAD itself was independently associated with significant disability (odds ratio 5.38; 95% confidence interval 3.78 to 7.64). Disability increased with CLAD stage (Figure 3). In CLAD stage 3 and 4, the prevalence was 50/120 (42%) in the BOS/other phenotype and 30/55 (55%) in the RAS or mixed phenotype. In CLAD stages 1 and 2, the prevalence was 12/71 (17%) in the BOS/other phenotype and 7/48 (15%) in the RAS or mixed phenotype.

The survival of transplants with CLAD and significant disability was clearly lower than that of patients without (median 4.04 years for patients with CLAD versus 5.41 years for patients without, *p* < 0.001), resulting in a difference of 1.37 years. As disability developed on average only after 2 years, patients with CLAD and significant disability lived for unadjusted 2.04 years with their restrictions before death (Table 3). When inserting the corresponding values into both equations, the YLL in CLAD patients were 1.288 years, and the YLD were 0.804 years. Consequently, the DALYs of those patients were on average 2.092 years. In sensitivity analysis for the DW, the YLD were 0.354 using the lower and 0.722 using the upper bound, resulting in DALYs of 1.99 and 2.71.

## 4. Discussion

To our knowledge, this is the first study analyzing disability in chronic lung allograft dysfunction. It is well known that the onset of CLAD is associated with impaired survival after lung transplantation in comparison to patients without CLAD. The exact number of life years lost due to CLAD is unknown, but mortality seems to be associated with the CLAD phenotype. In our study, a considerable fraction of 29% of all lung transplant patients developed CLAD, mostly with the BOS phenotype, whereby more than one-third developed significant disability. The calculated 2.09 DALYs (range 1.99–2.71) in this subgroup impressively demonstrate the impact of CLAD on lung transplant patients. Whilst the survival in unadjusted absolute numbers of CLAD patients with disability is already reduced by about 25% compared to patients without CLAD, nearly 10 adjusted months (0.804 years) are lost due to the restrictions of the disability itself. The impact of disability in our CLAD patients is probably underestimated as a minority (10%) of the 751 non-CLAD patients also developed significant disability. This was, however, not the focus of our evaluation.

The costs associated with lung transplantation are substantial. A 2019 study using US electronic medical records estimated the total costs of transplantation, including the transplant episode and one year of follow-up, as between USD 280,485 and USD 512,144 [15]. With respect to the subsequent development of CLAD, Sheshadri and coworkers estimate additional US costs due to inpatient admissions in years 1 and 2 following CLAD diagnosis of USD 99,372 and USD 83,348, respectively [16]. Consequently, each DALY associated with CLAD represents not only individual suffering but considerable economic losses, which in Germany have to be taken by the statutory health insurances.

It is well known that the onset of CLAD is associated with impaired survival after lung transplantation in comparison to patients without CLAD. The exact number of life years lost due to CLAD is unknown, but mortality seems to be associated with the CLAD phenotype [17].

Of note, according to our data, disability does not occur immediately but slowly develops within a median of 2 years after LTx. This fact offers options for the consistent drug prevention of CLAD. In a recently published study [18], Dellgren and coworkers demonstrate that tacrolimus-based immunosuppression once daily significantly reduced the incidence of CLAD compared to ciclosporine twice daily, and tacrolimus should be regarded as the first calcineurin inhibitor choice after LTx. Azithromycin has been shown to increase the BOS-free survival with a hazard ratio of 0.27 (95% CI 0.092–0.816). However, in contrast, there was no significant difference in overall survival with azithromycin in comparison to placebo [19].

This study has some limitations: First, it is a study at a single German center, and although a considerable number of patients were observed over a long period of time, it remains uncertain whether the results can be transferred to other countries. Second, only patients’ statements were considered in calculating significant disease, but the use of walking aids (wheelchair and rollator) was crosschecked by health care professionals using frailty scales. Third, we used an extended EuroQol 5D/5L questionnaire but otherwise followed Global Burden of Disease methods. However, our extension of the EuroQol has allowed us to approach as much as possible the criteria of severe COPD as defined by the GBD as a substitute for significant disability in CLAD patients. Fourth, some of our patients were selected at the beginning of the COVID-19 pandemic, and the last visit of 489 patients still coincided with the first six months after the pandemic. Thus, as SARS-CoV-2 infection in LTx patients may result in a significant decline in lung function and a higher mortality [20,21], it cannot be excluded that the results of our study might have been impaired by COVID-19. However, only one COVID-19 patient was included in the study cohort, and they survived the disease without disability.

There are also reports that SARS-CoV-2 mRNA vaccines in lung transplant recipients may potentially trigger the rejection and the development of CLAD [22]. In our LTx patients, however, the first vaccination was performed in January 2021, i.e., after the study period. In contrast, we have no information on the social distancing of those LTx patients who still had their rounds in the early months of the COVID-19 pandemic and may have benefited from a lower number of respiratory infections [23].

In conclusion, CLAD after lung transplantation is a major public health problem and is associated with substantial disability and costs. Further work is needed to develop therapeutic interventions that reduce its development.

## Figures and Tables

**Table 1:** Patient characteristics.

	*n* = 1025
Gender, female, n (%)	489 (48)
Age at transplant (years), median (25th, 75th percentile)	52.8 (40.0, 59.0)
Transplant procedure, n (%)	
Bilateral	983 (96)
Single lung	25 (2)
Heart–lung	17 (2)
Diagnosis, n (%)	
Fibrosis/interstitial lung disease	333 (33)
Chronic obstructive pulmonary disease/alpha1ATD	310 (30)
Cystic fibrosis/bronchiectasis	228 (22)
Pulmonary hypertension/pulmonary vascular diseases	67 (7)
Other	87 (9)
Median follow-up, months (25th, 75th percentile)	51 (27, 82)
Visits during follow-up, median (25th, 75th percentile)	17 (10, 25)
Death during follow-up, n (%)	212 (21)
Comorbidities during follow-up	
CLAD (persistent FEV < 80% baseline)	274 (27)
CLAD stage 3 or 4	117 (46)
CLAD phenotype BOS	157 (65)
CLAD phenotype RAS or mixed	70 (26)
CLAD phenotype undefined	14 (7)
Obesity (BMI > 30 kg/m^2^)	24 (2)
Significant kidney disease (GFR < 30 mL/min/1.73 m^2^)	47 (5)
Osteoporosis	27 (3)
Malignancy	23 (2)
Insulin-dependent diabetes	240 (23)
Coronary artery disease	51 (5)
Neuropathy	8 (1)
Stroke	9 (1)
Depression/psychosis	30 (3)
Peripheral arterial occlusive disease	10 (1)
Last Charlson comorbidity index, median	2 (1, 3)

ATD = antitrypsin deficiency; CLAD = chronic lung allograft dysfunction; BOS = bronchiolitis obliterans syndrome; RAS = restrictive allograft syndrome; BMI = body mass index; GFR = glomerular filtration rate.

**Table 2:** Multivariate analysis of predictors of significant disability.

Covariate	Significant Disability (Percentage in Brackets) n = 171 (17)	Significant Disability n = 854 (83)	Univariate		Multivariate	
			Odds Ratio (95% CI)	*p*	Odds Ratio (95% CI)	*p*
Gender						
Male	74 (14)	462 (86)	(Ref)	(Ref)	(Ref)	(Ref)
Female	97 (20)	392 (80)	1.545 (1.109, 2.151)	0.010	1.431 (0.998, 2.052)	0.051
Age at transplant, median years	53 (43, 58)	52 (39, 58)	1.007 (0.995, 1.020)	0.254		
Transplant procedure						
Bilateral	166 (17)	817 (83)	(Ref)	(Ref)		
Single lung	3 (12)	22 (88)	0.656 (0.149, 2.897)	0.578		
Heart–lung	2 (12)	15 (88)	0.671 (0.199, 2.268)	0.521		
Underlying disease						
Fibrosis/interstitial lung disease						
No	113 (16)	579 (84)	(Ref)	(Ref)		
Yes	58 (17)	275 (83)	1.081 (0.763, 1.530)	0.662		
COPD/alpha1ATD						
No	110 (15)	605 (85)	(Ref)	(Ref)	(Ref)	(Ref)
Yes	61 (20)	249 (80)	1.347 (0.953, 1.904)	0.091	1.247 (0.827, 1.879)	0.292
Cystic fibrosis/bronchiectasis						
No	146 (18)	651 (82)	(Ref)	(Ref)	(Ref)	(Ref)
Yes	25 (11)	203 (89)	0.549 (0.349, 0.863)	0.009	0.656 (0.392, 1.097)	0.108
PH/vascular disease						
No	153 (16)	805 (84)	(Ref)	(Ref)	(Ref)	(Ref)
Yes	18 (27)	49 (73)	1.933 (1.096, 3.408)	0.023	1.664 (0.862, 3.210)	0.129
Comorbidity						
CLAD						
No	72 (10)	679 (90)	(Ref)	(Ref)	(Ref)	(Ref)
Yes	99 (36)	175 (64)	5.335 (3.774, 7.541)	<0.001	5.375 (3.783, 7.639)	<0.001
BMI > 30 kg/m^2^						
No	167 (17)	834 (83)	(Ref)	(Ref)		
Yes	4 (17)	20 (83)	0.999 (0.337, 2.960)	0.998		
GFR < 30						
No	160 (16)	818 (84)	(Ref)	(Ref)		
Yes	11 (23)	36 (77)	1.562 (0.779, 3.134)	0.209		
Osteoporosis						
No	166 (17)	832 (83)	(Ref)	(Ref)		
Yes	5 (19)	22 (81)	1.139 (0.425, 3.051)	0.796		
Malignancy						
No	166 (17)	836 (83)	(Ref)	(Ref)		
Yes	5 (22)	18 (78)	1.399 (0.512, 3.821)	0.513		
Insulin-dependent diabetes						
No	131 (17)	654 (83)	(Ref)	(Ref)		
Yes	40 (17)	200 (83)	0.998 (0.677, 1.472)	0.994		
Coronary artery disease						
No	161 (17)	813 (83)	(Ref)	(Ref)		
Yes	10 (20)	41 (80)	1.232 (0.605, 2.509)	0.566		
Neuropathy						
No	170 (17)	847 (83)	(Ref)	(Ref)		
Yes	1 (12.5)	7 (87.5)	0.712 (0.087, 5.823)	0.751		
Stroke						
No	168 (17)	848 (83)	(Ref)	(Ref)		
Yes	3 (33)	6 (67)	2.524 (0.625, 10.191)	0.194		
Depression/psychosis						
No	162 (16)	833 (84)	(Ref)	(Ref)	(Ref)	(Ref)
Yes	9 (30)	21 (70)	2.204 (0.991, 4.899)	0.053	2.184 (0.922, 5.173)	0.076
Peripheral arterial occlusive disease						
No	170 (17)	845 (83)	(Ref)	(Ref)		
Yes	1 (10)	9 (90)	0.552 (0.070, 4.388)	0.574		

**Table 3:** Assumptions for calculating DALYs in CLAD patients with significant disability.

Age of death: 56.64 years (IQR 11.9)
Life expectancy at age of death years (compared to non-CLAD patients): 4.04
Discount rate: 0.03
Age weight: 0.04
Disability weight: 0.408 (0.354–0.722) [12]
Age of onset of disability: 54.6 years (IQR 22.2)
Duration of disability: 2.04 years (IQR 2.55)
Type of DALY used: definition of Murray 1996 [7]

## Data Availability

See Supplementary Materials.

## Supplementary Materials

The following supporting information can be downloaded at: https://www.mdpi.com/article/10.3390/arm91050033/s1, Table S1: Distribution of different groups of lung transplant patients to quality-of-life categories in the extended EuroQol 5D/5L questionnaire.
