# Supplementary material for: Chronic Lung Allograft Dysfunction Is Associated with Significant Disability after Lung Transplantation—A Burden of Disease Analysis in 1025 Cases
Source: Adv Respir Med. 2023 Oct 12;91(5):432–44. doi: 10.3390/arm91050033 (PMC10603923; doi:10.3390/arm91050033)
Supplement: Supplementary file 1 [file arm-91-00033-s001.zip › arm-2616188-supplementary.pdf]

Table S1. Distribution of different groups of lung transplant patients to quality-of-life categories in the extended EuroQol 5D/5L questionnaire

All patients	No CLAD	CLAD	BOS / undefined / unclassified	RAS / mixed
**Median EQ5D Score (n=909):** 95	**Median EQ5D Score (n=734):** 95	**Median EQ5D Score (n=175):** 85	**Median EQ5D Score (n=136):** 88	**Median EQ5D Score (n=39):** 75
**Mobility (n=915)**<br>No Problems 70.3%<br>Slight Problems 10.7%<br>Moderate Problems 15.7%<br>Severe Problems 3.1%<br>Unable 0.2%	**Mobility (n=737)**<br>No Problems 76.1%<br>Slight Problems 10.6%<br>Moderate Problems 11.0%<br>Severe Problems 2.2%<br>Unable 0.1%	**Mobility (n=178)**<br>No Problems 46.1%<br>Slight Problems 11.2%<br>Moderate Problems 35.4%<br>**Severe Problems 6.7%**<br>Unable 0.6%	**Mobility (n=137)**<br>No Problems 51.1%<br>Slight Problems 11.7%<br>Moderate Problems 29.2%<br>Severe Problems 7.3%<br>Unable 0.7%	**Mobility (n=41)**<br>No Problems 29.3%<br>Slight Problems 9.8%<br>Moderate Problems 56.1%<br>Severe Problems 4.9%<br>Unable 0.0%
**Self-care (n=914)**<br>No Problems 83.2%<br>Slight Problems 7.1%<br>Moderate Problems 8.4%<br>Severe Problems 0.8%<br>Unable 0.5%	**Self-care (n=736)**<br>No Problems 86.7%<br>Slight Problems 7.1%<br>Moderate Problems 5.8%<br>Severe Problems 0.3%<br>Unable 0.1%	**Self-care (n=178)**<br>No Problems 68.5%<br>Slight Problems 7.3%<br>Moderate Problems 19.1%<br>**Severe Problems 2.8%**<br>Unable 2.2%	**Self-care (n=137)**<br>No Problems 72.3%<br>Slight Problems 5.1%<br>Moderate Problems 17.5%<br>Severe Problems 2.9%<br>Unable 2.2%	**Self-care (n=41)**<br>No Problems 56.1%<br>Slight Problems 14.6%<br>Moderate Problems 24.4%<br>Severe Problems 2.4%<br>Unable 2.4%
**Usual Activities (n=912)**<br>No Problems 67.0%<br>Slight Problems 14.5%<br>Moderate Problems 14.9%<br>Severe Problems 2.0%<br>Unable 1.6%	**Usual Activities (n=735)**<br>No Problems 71.6%<br>Slight Problems 15.0%<br>Moderate Problems 11.4%<br>Severe Problems 1.4%<br>Unable 0.7%	**Usual Activities (n=177)**<br>No Problems 48.0%<br>Slight Problems 12.4%<br>Moderate Problems 29.4%<br>Severe Problems 4.5%<br>Unable 5.6%	**Usual Activities (n=137)**<br>No Problems 50.4%<br>Slight Problems 11.7%<br>Moderate Problems 27.7%<br>Severe Problems 5.8%<br>Unable 4.4%	**Usual Activities (n=40)**<br>No Problems 40.0%<br>Slight Problems 15.0%<br>Moderate Problems 35.0%<br>Severe Problems 0.0%<br>Unable 10.0%
**Pain / Discomfort (n=914)**<br>No Pain / Discomfort 54.5%<br>Slight Pain / Discomfort 22.2%<br>Moderate Pain / Discomfort 17.8%<br>Severe Pain / Discomfort 4.0%<br>Extreme Pain / Discomfort 1.4%	**Pain / Discomfort (n=736)**<br>No Pain / Discomfort 55.7%<br>Slight Pain / Discomfort 23.8%<br>Moderate Pain / Discomfort 15.8%<br>Severe Pain / Discomfort 3.5%<br>Extreme Pain / Discomfort 1.2%	**Pain / Discomfort (n=178)**<br>No Pain / Discomfort 49.4%<br>Slight Pain / Discomfort 15.7%<br>Moderate Pain / Discomfort 26.4%<br>Severe Pain / Discomfort 6.2%<br>Extreme Pain / Discomfort 2.2%	**Pain / Discomfort (n=137)**<br>No Pain / Discomfort 51.8%<br>Slight Pain / Discomfort 15.3%<br>Moderate Pain / Discomfort 24.1%<br>Severe Pain / Discomfort 7.3%<br>Extreme Pain / Discomfort 1.5%	**Pain / Discomfort (n=41)**<br>No Pain / Discomfort 41.5%<br>Slight Pain / Discomfort 17.1%<br>Moderate Pain / Discomfort 34.1%<br>Severe Pain / Discomfort 2.4%<br>Extreme Pain / Discomfort 4.9%
**Anxiety / Depression (n=911)**<br>Not anxious or depressed 76.1%<br>Slightly anxious or depressed 14.1%<br>Moderately anxious or depressed 8.7%<br>Severely anxious or depressed 0.8%<br>Extremely anxious or depressed 0.4%	**Anxiety / Depression (n=735)**<br>Not anxious or depressed 78.6%<br>Slightly anxious or depressed 14.0%<br>Moderately anxious or depressed 6.5%<br>Severely anxious or depressed 0.4%<br>Extremely anxious or depressed 0.4%	**Anxiety / Depression (n=176)**<br>Not anxious or depressed 65.3%<br>Slightly anxious or depressed 14.2%<br>Moderately anxious or depressed 17.6%<br>**Severely anxious or depressed 2.3%**<br>Extremely anxious or depressed 0.6%	**Anxiety / Depression (n=136)**<br>Not anxious or depressed 67.6%<br>Slightly anxious or depressed 12.5%<br>Moderately anxious or depressed 17.6%<br>Severely anxious or depressed 2.2%<br>Extremely anxious or depressed 0.0%	**Anxiety / Depression (n=40)**<br>Not anxious or depressed 57.5%<br>Slightly anxious or depressed 20.0%<br>Moderately anxious or depressed 17.5%<br>Severely anxious or depressed 2.5%<br>Extremely anxious or depressed 2.5%
**Flights of Stairs (n=1022)**<br>> 2 Flights of Stairs 48.4%<br>2 Flights of Stairs 22.9%<br>1 Flights of stairs 19.6%<br>No Flights of stairs 9.1%	**Flights of Stairs (n=782)**<br>> 2 Flights of Stairs 57.5%<br>2 Flights of Stairs 23.5%<br>1 Flights of stairs 14.8%<br>No Flights of stairs 4.1%	**Flights of Stairs (n=240)**<br>> 2 Flights of Stairs 18.8%<br>2 Flights of Stairs 20.8%<br>1 Flights of stairs 35.0%<br>No Flights of stairs 25.4%	**Flights of Stairs (n=170)**<br>> 2 Flights of Stairs 24.4%<br>2 Flights of Stairs 20.6%<br>1 Flights of stairs 33.5%<br>No Flights of stairs 21.8%	**Flights of Stairs (n=70)**<br>> 2 Flights of Stairs 5.7%<br>2 Flights of Stairs 21.4%<br>1 Flights of stairs 38.6%<br>No Flights of stairs 34.3%
**Rollator/wheelchair (n=1023)**<br>No 94.7%<br>Yes 5.3%	**Rollator/wheelchair (n=782)**<br>No 96.7%<br>Yes 3.3%	**Rollator/wheelchair (n=241)**<br>No 88.4%<br>**Yes 11.6%**	**Rollator/wheelchair (n=171)**<br>No 90.6%<br>Yes 9.4%	**Rollator/wheelchair (n=70)**<br>No 82.9%<br>Yes 17.1%
